# Downregulation of LncRNA-XIST inhibited development of non-small cell lung cancer by activating miR-335/SOD2/ROS signal pathway mediated pyroptotic cell death

**DOI:** 10.18632/aging.102291

**Published:** 2019-09-25

**Authors:** Jinglei Liu, Lei Yao, Mingyan Zhang, Ji Jiang, Maopeng Yang, Yue Wang

**Affiliations:** 1 Harbin Medical University, Heilongjiang, China; 2 Department of Medical Oncology, The Third Affiliated Hospital of Harbin Medical University, Heilong Jiang, China; 3 Department of Thoracic Surgery, The Third Affiliated Hospital of Harbin Medical University, Heilongjiang, China; 4 Department of Laboratory, The Third Affiliated Hospital of Harbin Medical University, Heilongjiang, China; 5 Department of nuclear medicine, The Third Affiliated Hospital of Harbin Medical University, Heilongjiang, China; 6 Department of Pharmacology and Toxicology, Wright State University, Dayton, OH 45435, USA

**Keywords:** LncRNA-XIST, NSCLC, miR-335, SOD2, pyroptosis

## Abstract

LncRNA-XIST participated in the regulation of Non-small cell lung cancer (NSCLC) progression, but the underlying mechanisms are still unclear. This study showed that LncRNA-XIST aberrantly overexpressed in either NSCLC tissues or cell lines comparing to their paired control groups. Knock-down of LncRNA-XIST promoted NSCLC cell apoptosis and inhibited cell proliferation, which were reversed by synergistically treating cells with pyroptosis inhibitor Necrosulfonamide (NSA). In addition, knock-down of LncRNA-XIST also promoted reactive oxygen species (ROS) production and NLRP3 inflammasome activation. In parallel, ROS scavenger N-acetyl cysteine (NAC) abrogated the effects of downregulated LncRNA-XIST on NSCLC cell pyroptosis. Furthermore, miR-335 was the downstream target of LncRNA-XIST and overexpressed LncRNA-XIST increased SOD2 expression levels by sponging miR-335. Mechanistically, miR-335 inhibitor reversed the effects of downregulated LncRNA-XIST on ROS levels and cell pyroptosis, which were abrogated by synergistically knocking down SOD2. Taken together, knock-down of LncRNA-XIST inhibited NSCLC progression by triggering miR-335/SOD2/ROS signal pathway mediated pyroptotic cell death.

## INTRODUCTION

Non-small cell lung cancer (NSCLC) is one of the most common lung associated cancer [1], which seriously endangered the health of human beings. However, there are still no effective therapies for NSCLC treatment in clinic [2], uncovering the underlying mechanisms of NSCLC pathogenesis might solve this problem. Long-noncoding RNAs (lncRNAs) was closely related with the development of multiple cancers, such as bladder cancer [3], gastric cancer [4], ovarian cancer [5] and NSCLC [6]. For example, lncRNA LINC-PINT inhibited NSCLC progression by targeting miR-218-5p/PDCD4 [6] and LncRNA AWPPH promoted NSCLC cell proliferation by activating Wnt/β-catenin signaling pathway [7]. LncRNA-XIST served as an oncogene in various cancers [8, 9] and promoted NSCLC development [10–12]. For example, lncRNA-XIST promoted NSCLC cell proliferation, invasion and metastasis [13].

LncRNAs regulated cell functions by targeting microRNAs (miRNAs) [14, 15]. For example, LncRNA-XIST promoted TGF-β-induced epithelial-mesenchymal transition (EMT) of NSCLC cells by targeting miR-367 [10]. Besides, LncRNA-XIST promoted NSCLC cell proliferation and invasion by sponging miR-186-5p [13]. The online starBase software (http://starbase.sysu.edu.cn/index.php) [16] predicted that miR-335 was the downstream target of LncRNA-XIST, which was further validated by our dual-luciferase reporter gene system. In addition, miR-335 was a tumor suppressor and inhibited the development of NSCLC [17–19]. Of note, miR-335 was closely related with oxidative stress [20, 21]. For example, miR-335 promoted oxidative stress-mediated aging of endothelial cells [21]. Notably, mitochondrial antioxidative enzyme SOD2 could be suppressed by miR-335 in renal senescence [20].

Pyroptotic cell death, also known as pyroptosis or inflammatory cell necrosis, which was mediated by gasdermin family and accompanied by inflammatory response [22]. Recent studies suggested that triggering pyroptotic cell death might shed light on cancer treatment in clinic [22–24]. Nucleotide-binding domain leucine-rich repeats family protein 3 (NLRP3) inflammasome activation was the key procedure of cell pyroptosis [25, 26]. Researchers found that LncRNA-XIST [27] and miR-353 [28] could regulate NLRP3 inflammsome activation. Specifically, LncRNA-XIST mediated bovine mammary epithelial cell inflammatory reactions by inhibiting NLRP3 inflammasome activation [27]. Besides, miR-335 activated NLRP3 inflammasome in intervertebral disk degeneration [28]. Furthermore, cell pyroptosis could be induced by oxidative stress [29, 30]. For example, mitochondrial ROS promoted macrophage pyroptotic cell death by inducing GSDMD oxidation [29] and ROS could induce cell pyroptotic cell death in pancreatic cancer progression [30].

Based on the above studies, we hypothesized that LncRNA-XIST might be involved in NSCLC progression by targeting miR-335/SOD2 signal pathway. In addition, oxidative stress and pyroptosis might also be crucial for NSCLC development. This study will provide potential therapeutic agents for NSCLC treatment in clinic.

## RESULTS

### The expression levels of LncRNA-XIST in NSCLC tissues and cell lines

**Figure 1:**
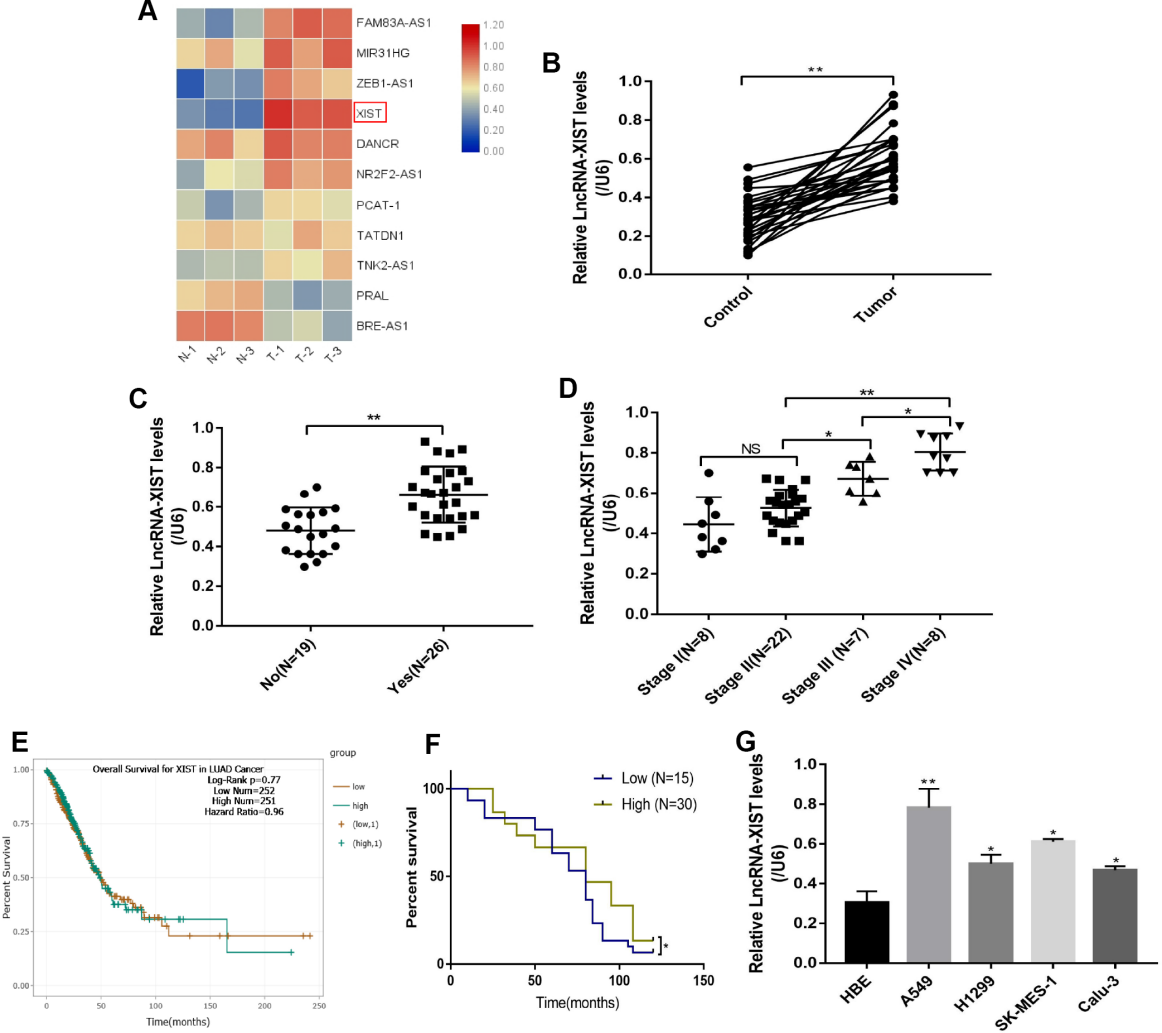
**The correlation between LncRNA-XIST expression levels and NSCLC development.** (**A**) Real-Time qPCR was used to screen NSCLC associated LncRNAs. (**B**) Real-Time qPCR was used to detect LncRNA-XIST levels in 30 paired clinical tissues collected from NSCLC patients. (**C**, **D**) LncRNA-XIST levels were compared according to lymphatic metastasis (**C**) and TNM stage. (**E**) Pan-cancer analysis was conducted to investigate the correlation of LncRNA-XIST levels and prognosis of patients with lung adenocarcinoma. (**F**) Kaplan-Meier analysis was used to analyse the percent survival of NSCLC patients in our experiments. (**J**) Real-Time qPCR was used to detect LncRNA-XIST levels in HBE, A549, H1299, SK-MES-1 and Calu-3 cell lines. (“NS” represented no statistical significance, “*” represented *p* < 0.05, “**” represented *p* < 0.01).

We first screened the expression levels of several common NSCLC associated LncRNAs in NSCLC tissues collected from clinic (Figure 1A). The results showed that LncRNA-XIST was most significantly overexpressed in NSCLC tissues comparing to the adjacent normal tissues (Figure 1A), which were further validated by the Real-Time qPCR results from additional 30 NSCLC patients (Figure 1B). In addition, by analysing the correlation of LncRNA-XIST levels with different clinical parameters (Age, Gender, TNM Stage, Pathological Type, Smoking status and Lymphatic metastasis), we found that LncRNA-XIST levels has nothing to do with patients age (Supplementary Figure 1B), gender (Supplementary Figure 1C), smoking status (Supplementary Figure 1A) and pathological type (Supplementary Figure 1D). Interestingly, the results showed that LncRNA-XIST levels were positively correlated with TNM stage (Figure 1D) and lymphatic metastasis (Figure 1C). The Pan-cancer analysis results showed that lung adenocarcinoma patients with higher LncRNA-XIST levels had comparatively worse prognosis and shorter survival time eventhough without statistical significance (Figure 1E), further Kaplan-Meier analysis validated that the percent survival was significantly higher in patients with low-expressed LncRNA-XIST comparing to their counterparts (Figure 1F). Furthermore, the NSCLC cell lines (A549, H1299, SK-MES-1 and Calu-3) and human bronchial epithelial cell line (HBE) were selected to explore the aberrant expression of LncRNA-XIST in cellular levels (Figure 1G). The results showed that the levels of LncRNA-XIST in A549, H1299 and SK-MES-1 and Calu-3 were significantly higher than HBE cells (Figure 1G). As LncRNA-XIST was low-expressed in H1299 cells comparing to A549 cells, we chose to knock down LncRNA-XIST in A549 cells and overexpress LncRNA-XIST in H1299 cells for further experiments.

### Influences of LncRNA-XIST on NSCLC cell proliferation and apoptosis

**Figure 2:**
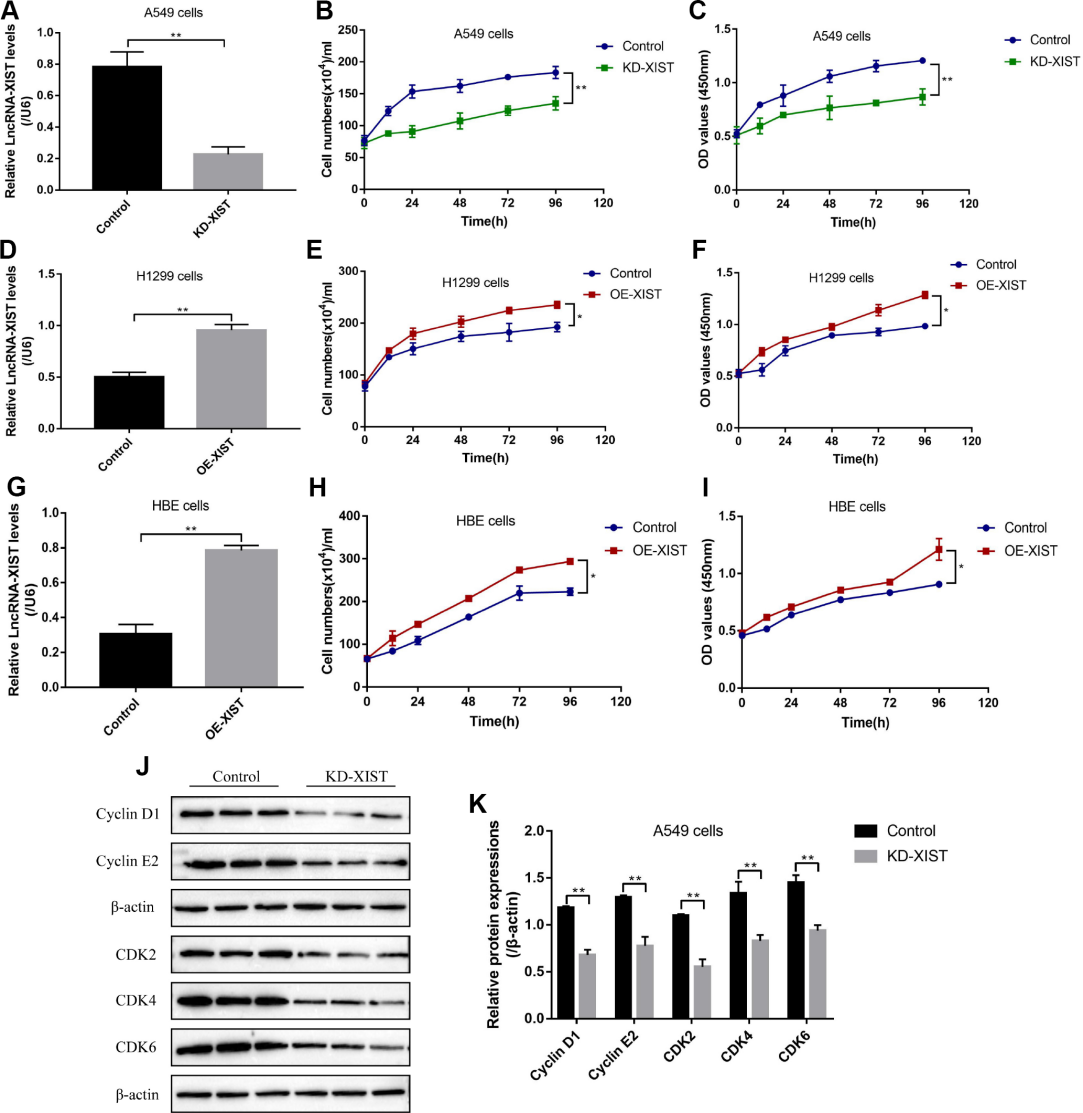
**The effects of LncRNA-XIST on NSCLC cell proliferation.** (**A**) Real-Time qPCR was used to detect LncRNA-XIST levels in A549 cells. (**B**) Cell counting assay was used to count A549 cell numbers. (**C**) CCK-8 kit was used to evaluate A549 cell proliferation. (**D**) Real-Time qPCR was used to detect LncRNA-XIST levels in H1299 cells. (**E**) Cell counting assay was used to count H1299 cell numbers. (**F**) CCK-8 kit was used to evaluate H1299 cell proliferation. (**G**) Real-Time qPCR was used to detect LncRNA-XIST levels in HBE cells. (**H**) Cell counting assay was used to count HBE cell numbers. (**I**) CCK-8 kit was used to evaluate HBE cell proliferation. (**J**) Western Blot was used to detect cell cycle associated proteins (Cyclin D1, Cyclin E2, CDK2, CDK4 and CDK6), which was normalized to β-actin and (**K**) quantified by Image J software. (“NS” represented no statistical significance, “*” represented *p* < 0.05, “**” represented *p* < 0.01).

**Figure 3:**
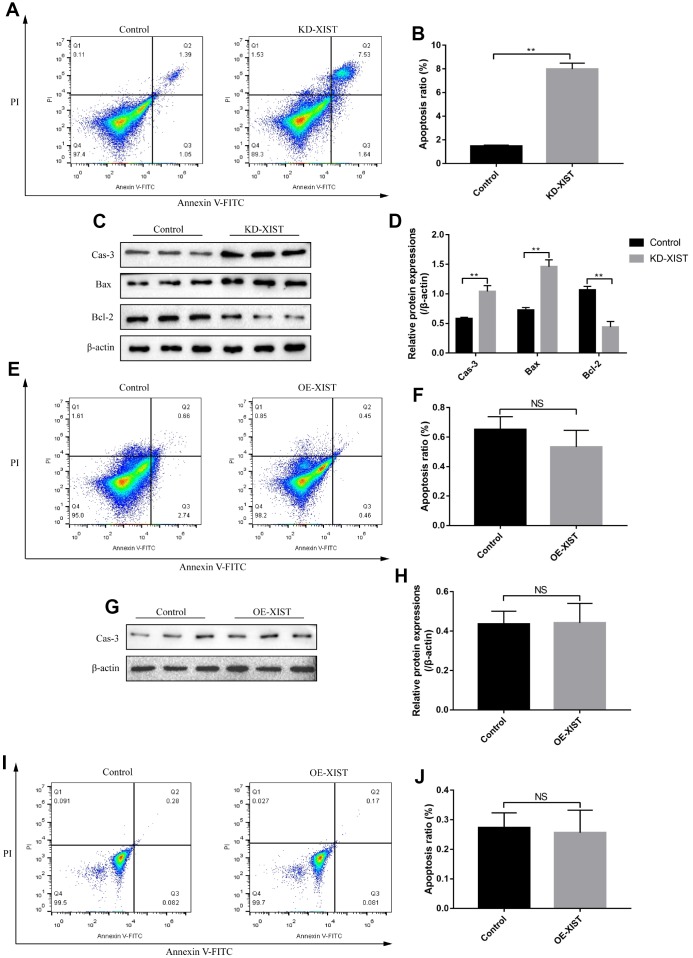
**The effects of LncRNA-XIST on NSCLC cell apoptosis.** (**A**) FCM was used to detect A549 cell apoptosis and (**B**) quantification was conducted. (**C**) The expression levels of apoptosis associated proteins (Caspase-3, Bax and Bcl-2) in A549 cells, which were normalized to β-actin and (**D**) quantified by Image J software. (**E**) FCM was used to detect H1299 cell apoptosis and (**F**) quantification was conducted. (**G**) Western Blot was used to detect Caspase-3 levels in H1299 cells, which were normalized to β-actin and (**H**) quantified by Image J software. (**I**, **J**) The apoptosis ratio of HBE cells was detected by FCM. (“NS” represented no statistical significance, “*” represented *p* < 0.05, “**” represented *p* < 0.01).

To investigate the effects of LncRNA-XIST on NSCLC and HBE cell functions, the sh-LncRNA-XIST were transfected into A549 cells and overexpressed vectors were transfected into H1299 and HBE cells. The results showed that we have successfully established the downregulated LncRNA-XIST A549 cell models and overexpressed LncRNA-XIST H1299 and HBE cell models respectively (Figure 2A, 2D, 2G). The cell counting assay and CCK-8 results showed that knock-down of LncRNA-XIST inhibited A549 cell proliferation (Figure 2B, 2C) and overexpressed LncRNA-XIST promoted H1299 (Figure 2E, 2F) and HBE cell proliferation (Figure 2H, 2I). The Western Blot results also showed that the cell cycle associated proteins including Cyclin D1, Cyclin E2, CDK2, CDK4 and CDK6 were downregulated by knocking down LncRNA-XIST in A549 cells (Figure 2J, 2K). In parallel, the FCM results showed that knock-down of LncRNA- XIST increased A549 cell apoptosis ratio (Figure 3A, 3B). In addition, downregulated LncRNA-XIST increased pro-apoptotic proteins (Caspase-3 and Bax) and decreased anti-apoptotic protein Bcl-2 in A549 cells (Figure 3C, 3D). However, our results showed that overexpressed LncRNA-XIST has little effects on cell apoptosis ratio (Figure 3E, 3F) and Caspase 3 levels (Figure 3G, 3H) in H1299 cells. Similarly, overexpressed LncRNA-XIST has little effects on HBE cell apoptosis (Figure 3I, 3J).

### LncRNA-XIST influenced NSCLC cell viability by regulating ROS-induced pyroptotic cell death

**Figure 4:**
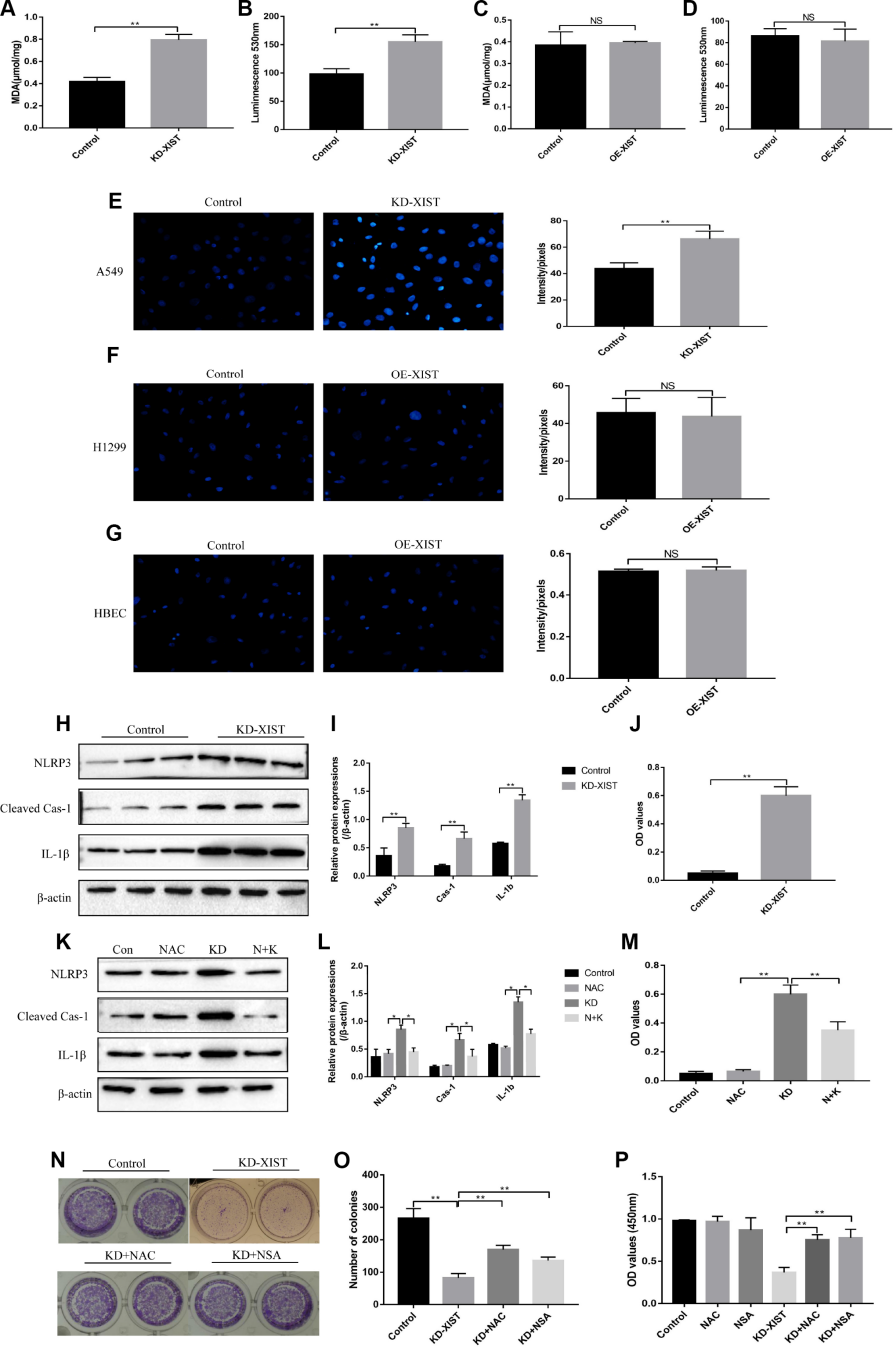
**The influences of LncRNA-XIST on oxidative stress and pyroptosis of NSCLC cell lines.** MDA levels was detected in (**A**) A549 cells and (**C**) H1299 cells. Extracellular NADPH oxidase-derived superoxide was detected in (**B**) A549 cells and (**D**) H1299 cells. DHE staining was used to detect ROS levels in (**E**) A549 cells, (**F**) H1299 cells and (**G**) HBE cells. (**H**, **K**) Western Blot was used to detect pyroptosis associated proteins (NLRP3, Cleaved Caspase-1 and IL-1β), which was normalized to β-actin and (**I**, **L**) quantified by Image J software. (**J**, **M**) ELSIA kit was used to detect IL-18 levels in A549 cell supernatants. (**N**, **O**) Colony formation assay was performed to detect cell proliferative abilities. (**P**) CCK-8 assay was used to detect A549 cell proliferation. “NAC” means N-acetyl cysteine treatment. “KD” means knock-down of LncRNA-XIST. “N+K” means NAC treatment plus knocking down LncRNA-XIST. (“NS” represented no statistical significance, “*” represented *p* < 0.05, “**” represented *p* < 0.01).

Interestingly, our results showed that knock-down of LncRNA-XIST increased MDA as well as ROS levels (Figure 4A, 4E), and promoted superoxide release (Figure 4B) in A549 cells, while overexpressed LncRNA-XIST could not affect oxidative stress in H1299 (Figure 4C, 4D, 4F) and HBE cells (Figure 4G). Further results showed that downregulated LncRNA-XIST activated NLRP3 inflammasome and increased cleaved Caspase-1 as well as mature IL-1β in A549 cells (Figure 4H, 4I). Besides, IL-18 levels were also increased by knocking down LncRNA-XIST (Figure 4J). Previous studies have proved that oxidative stress was closely related with cell pyroptosis [29] and ROS induced cell pyroptosis by activating NLRP3 inflammasome [31]. To further validate whether LncRNA-XIST affected cell pyroptosis by regulating ROS generation, the ROS scavenger N-acetyl cysteine (NAC) was used to treat A549 cells. The results showed that NAC treatment reversed the effects of downregulated LncRNA-XIST on A549 cell pyroptosis (Figure 4K, 4L) and cell proliferation (Figure 4M). Furthermore, the Colony formation assay and CCK-8 results showed that the inhibiting effects of downregulated LncRNA-XIST on A549 cell proliferative abilities were abrogated by either NAC or pyroptosis inhibitor NSA (Figure 4N–4P).

### Downregulation of LncRNA-XIST affected ROS-induced pyroptotic cell death by regulating SOD2

**Figure 5:**
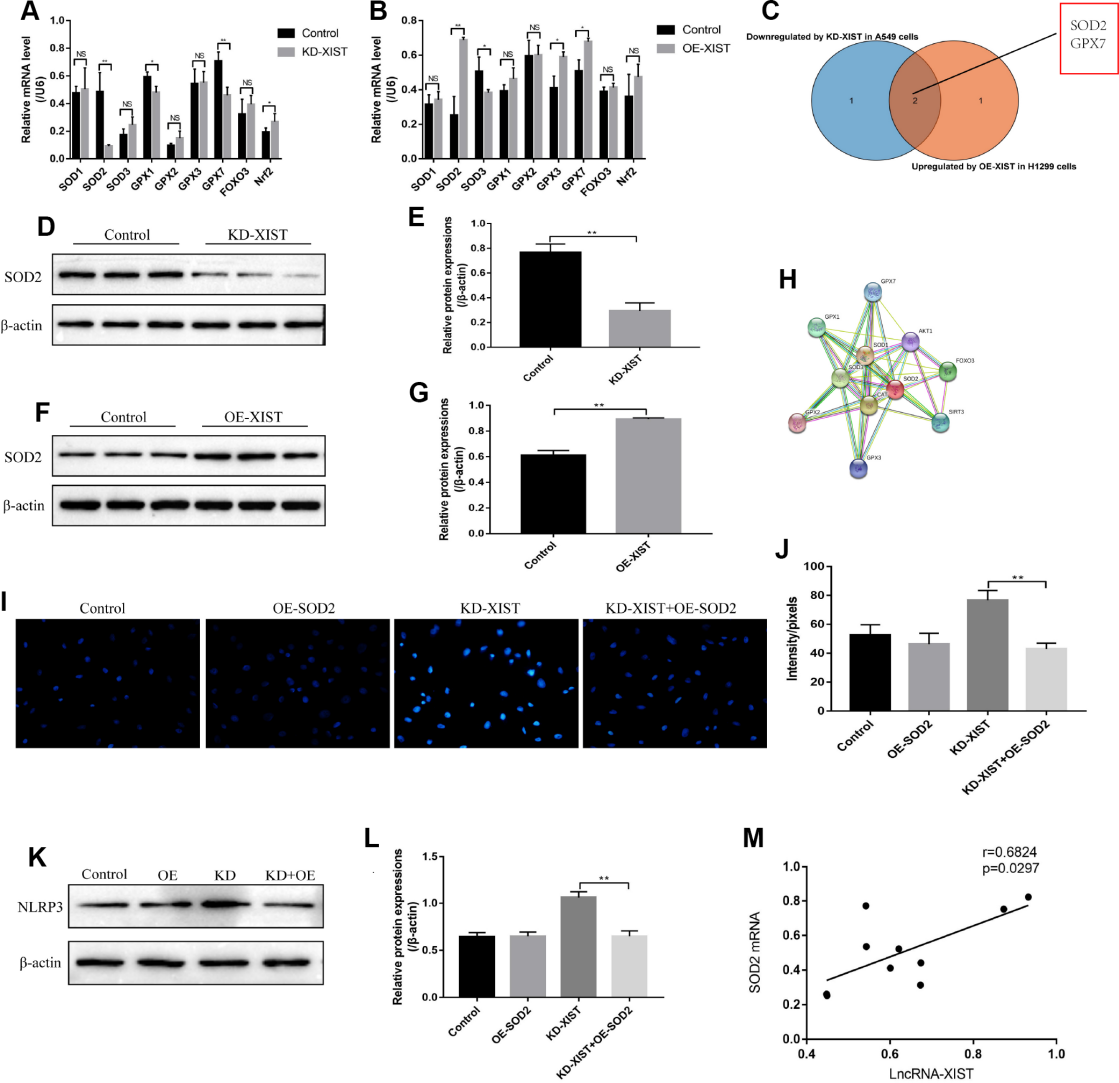
**The role of SOD2 in LncRNA-XIST regulated cell pyroptosis and ROS generation.** (**A**) Real-Time qPCR was used to detect the levels of anti-oxidant genes in A549 cells. (**B**) Real-Time qPCR was used to detect the levels of anti-oxidant genes in H1299 cells. (**C**) Venn diagram showing the overlapped genes downreglated in A549 cells and upregulated in H1299 cells. Western Blot was used to detect SOD levels in (**D**) A549 cells and (**F**) H1299 cells, which were normalized to β-actin and (**E**, **G**) quantified by Image J software. (**H**) STRING software was used to conduct co-expression analysis of SOD2. (**I**, **J**) DHE staining was used to detect ROS levels in A549 cells. (**K**) NLRP3 levels were detected by Western Blot in A549 cells, which were normalized to β-actin and (**L**) quantified by Image J software. (**M**) Pearson analysis was conducted to analyse the correlation between LncRNA-XIST and SOD2 mRNA levels tin NSCLC tissues. (“NS” represented no statistical significance, “*” represented *p* < 0.05, “**” represented *p* < 0.01).

Since oxidative stress could be regulated by multiple gene alterations, hence we next explored the possible involved genes. Real-Time qPCR was used to screen the differential expressions of anti-oxidant genes (SOD1, SOD2, SOD3, GPX1, GPX2, GPX3, GPX7, FOXO3 and Nrf2) in NSCLC cells deficient or upregulation of LncRNA-XIST (Figure 5A, 5B). Further results showed that the levels of LncRNA-XIST and SOD2 mRNA were positively correlated (Figure 5M). The results showed that the mRNA levels of SOD2 was significantly downregulated by knocking down LncRNA-XIST in A549 cells and upregulated by overexpressed LncRNA-XIST in H1299 cells (Figure 5A–5C). In parallel, the Western Blot results validated that SOD2 levels was downregulated by knocking down LncRNA-XIST in A549 cells (Figure 5D, 5E), and overexpressed LncRNA-XIST upregulated SOD2 levels in H1299 cells (Figure 5F, 5G). Of note, we found that overexpressed SOD2 abrogated the effects of downregulated LncRNA-XIST on A549 cell ROS production (Figure 5I, 5J) and pyroptosis (Figure 5K, 5L). The co-expression analysis by the STRING software showed that SOD2 was closely related with oxidative stress related genes such as glutathione peroxidase 1 (GPX1), Catalase (CAT), Forkhead box protein O3 (FOXO3) and so on (Figure 5H).

### LncRNA-XIST regulated SOD2 levels by targeting miR-335

**Figure 6:**
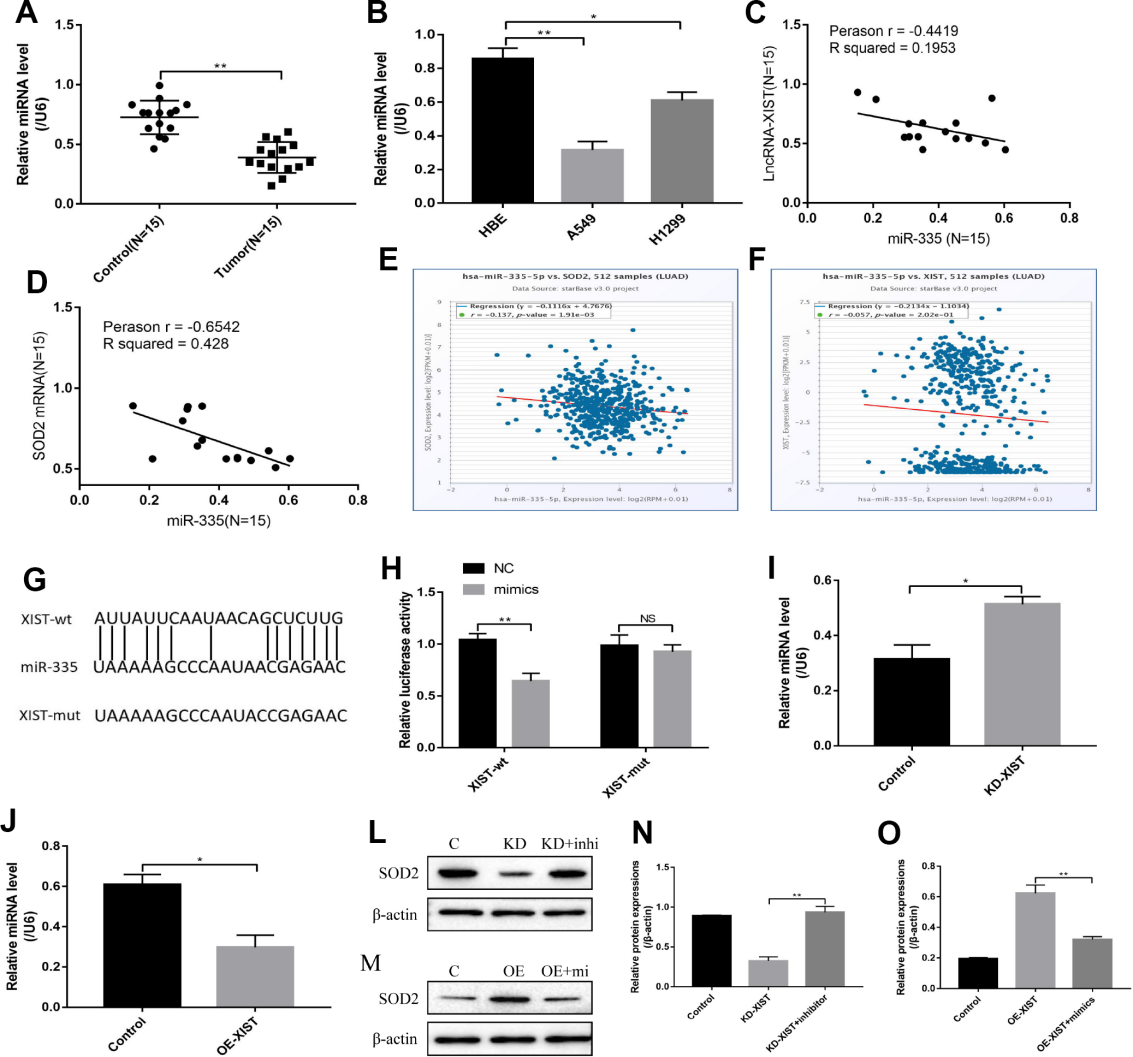
**LncRNA-XIST regulated SOD2 levels by targeting miR-335.** (**A**) Real-Time qPCR was used to detect miR-335 levels in NSCLC tissues. (**B**) miR-335 levels were detected in NSCLC cell lines. (**C**) Pearson analysis was used to evaluate correlation of miR-335 and LncRNA-XIST in NSCLC tissues (N=15). (**D**) Pearson analysis was used to evaluate correlation of miR-335 and SOD2 mRNA levels in NSCLC tissues (N=15). (**E**, **F**) Pan-cancer analysis was used to analyse the correlation of LncRNA-XIST, miR-335 and SOD2 expression levels in lung adenocarcinoma. (**G**) Sequence alignment of miR-335 with the putative binding sites with in the wild-type regions of LncRNA-XIST. (**H**) Dual-luciferase reporter gene system was conducted to investigate the targeting relationship between LncRNA-XIST and miR-335. (**I**, **J**) The effects of LncRNA-XIST on miR-335 were detected by Real-Time qPCR in A549 and H1299 cells respectively. (**L**, **N**) Knock-down (KD) of LncRNA-XISt regulated SOD2 levels in A549 cells by targeting miR-335. (**M**, **O**) Overexpressed (OE) LncRNA-XIST regulated SOD2 levels in H1299 cells by targeting miR-335. (“NS” represented no statistical significance, “*” represented *p* < 0.05, “**” represented *p* < 0.01).

Previous study has reported that SOD2 was the down stream target of miR-335 [20], and the online starBase software predicted that miR-335 was the downstream target of LncRNA-XIST (Figure 6G). Our results showed that miR-335 was downregulated in either NSCLC tissues or cell lines comparing to the Control group (Figure 6A, 6B), which was negatively correlated with LncRNA-XIST and SOD2 (Figure 6C, 6D) and in line with the Pan-cancer analysis in lung adenocarcinoma (Figure 6E, 6F). Since we have proved that LncRNA-XIST regulated SOD2 levels, it was reasonable to speculate that LncRNA-XIST might regulate SOD2 by targeting miR-335. The results showed that Knock- down of LncRNA-XIST increased miR-335 levels in A549 cells (Figure 6I). In parallel, overexpressed LncRNA-XIST inhibited miR-335 levels in H1299 cells (Figure 6J). The dual-luciferase reporter gene system results showed that miR-335 mimic decreased luciferase activity by binding to the 3′-Untranslated region (UTR) of LncRNA-XIST (Figure 6H). Furthermore, we found that miR-335 inhibitors restored SOD2 expression levels in LncRNA-XIST knock-down A549 cells (Figure 6L, 6N). Similarly, miR-335 mimic abrogated the promoting effects of overexpressed LncRNA-XIST on SOD2 in H1299 cells (Figure 6M, 6O).

### Knock-down of LncRNA-XIST inhibited A549 cell viability and promoted cell apoptosis by regulating miR-335/SOD2 signal pathway

**Figure 7:**
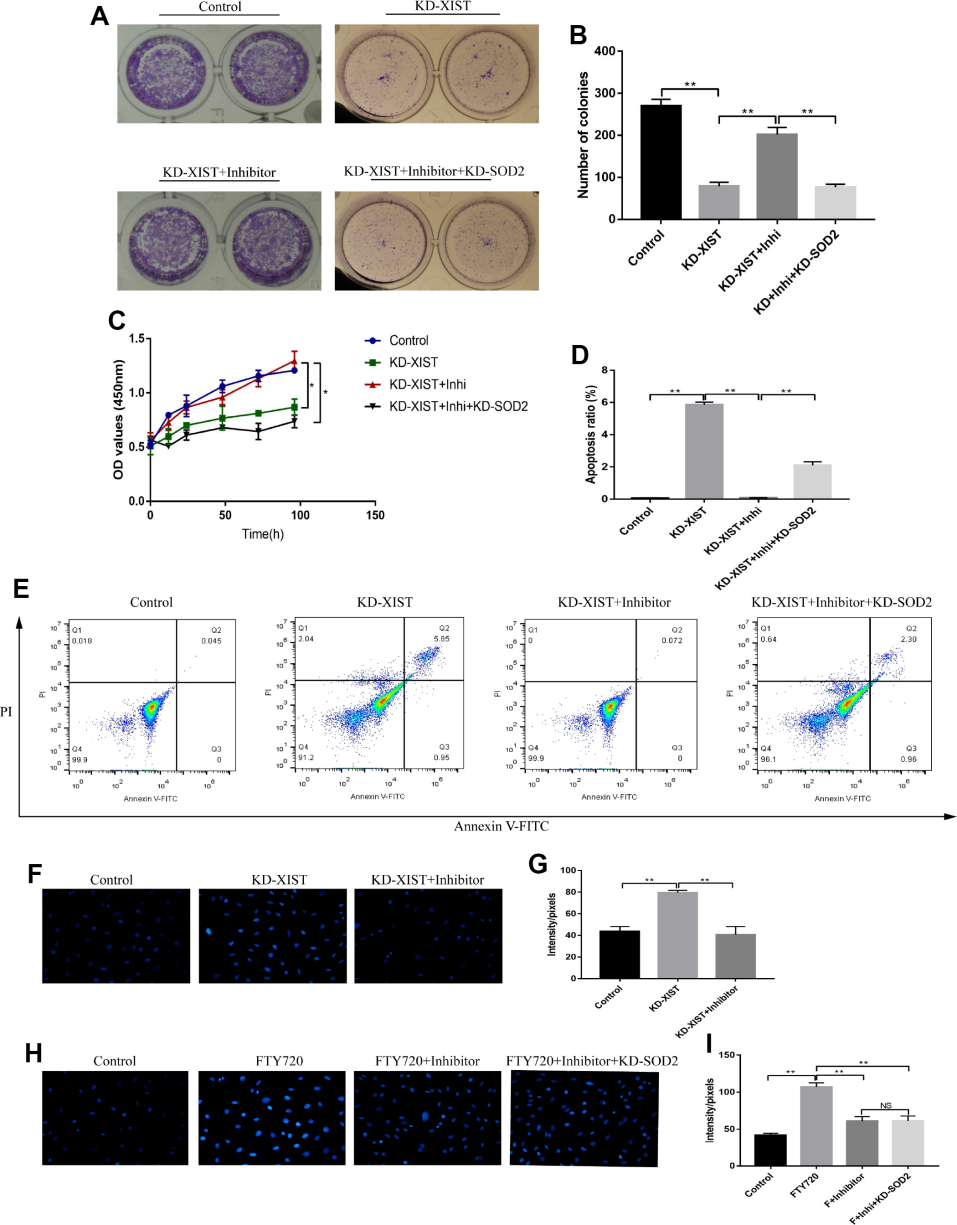
**The effects of LncRNA-XIST on A549 cell proliferation and apoptosis by targeting miR-335/SOD2 axis.** (**A**, **B**) Colony formation assay and (**C**) CCK-8 assay were used to detect A549 cell proliferation. (**D**, **E**) The apoptosis ratio of A549 cells was quantified by FCM. (**F**, **H**) ROS levels in A549 cells were detected by DHE staining, which were quantified by (**G**, **I**) Image J software. (“NS” represented no statistical significance, “*” represented *p* < 0.05, “**” represented *p* < 0.01).

We next investigated whether knock-down of LncRNA-XIST inhibited NSCLC cell viability by targeting miR-335/SOD2 signal pathway. The Colony formation assay and CCK-8 results showed that miR-335 inhibitor reversed the inhibiting effects of downregulated LncRNA-XIST on cell proliferation, which was abrogated by synergistically knocking down SOD2 (Figure 7A–7C). Besides, we found that knock-down of LncRNA-XIST also affected cell apoptosis by targeting miR-335/SOD signal pathway in a similar way (Figure 7D, 7E). Specifically, miR-335 inhibitor reversed the promoting effects of downregulated LncRNA-XIST on cell apoptosis, which were abrogated by synergistically knocking down SOD2 (Figure 7D, 7E). In addition, knock-down of LncRNA-XIST increased ROS levels in A549 cells, which was reversed by synergistically treating cells with miR-335 inhibitor (Figure 7F, 7G). In addition, we found that miR-335 inhibitor successfully inhibited ROS levels induced by fingolimod hydrochloride (FTY720), a ROS inducer has been proved in the previous study [32], in NSCLC cell lines. However, the effects of miR-335 inhibitor on FTY720 induced ROS generation were not reversed by knocking down SOD2 in NSCLC cells (Figure 7H, 7I), which indicated that FTY720 might regulate oxidative stress in NSCLC cells by targeting miR-335 in a SOD2 independent way. However, the detailed mechanisms are still need to be elucidated.

## DISCUSSION

NSCLC has seriously endangered the health of human beings [1], however, as the results of the complexity of NSCLC pathogenesis, there are still no effective treatment for NSCLC in clinic [2]. Uncovering the underlying mechanisms will help to provide new therapeutic agents for NSCLC treatment. Recent studies have reported that LncRNAs played an important role in NSCLC pathogenesis [6, 7]. By screening multiple common NSCLC associated LncRNAs, we identified that LncRNA-XIST was high-expressed in NSCLC tissues and cell lines comparing to the adjacent normal tissues and HBE cells respectively. In addition, the levels of LncRNA-XIST was positively related with TNM stage and lymphatic metastasis. Eventhough we have determined the aberrant expressions of LncRNA-XIST in NSCLC specimens, it is still unclear the localization of LncRNA-XIST in NSCLC microenvironment. Therefore, the in situ hybridization is still need to be conducted to localize LncRNA-XIST in NSCLC tissues in our further experiments.

Further results showed that NSCLC cell proliferation and apoptosis could be regulated by downregulating LncRNA-XIST. However, overexpressed LncRNA-XIST merely promoted cell proliferation, but had little effects on cell apoptosis, which might be due to the low apoptosis ratio of NSCLC cells in the standard culture conditions. Mechanistically, NSCLC cells might need to sustain LncRNA-XIST in a certain levels to guarantee their biological functions, hence downregulated instead of overexpressed LncRNA-XIST had significant impacts on NSCLC cell viability, but the detailed mechanisms are still need to be elucidated in our further experiments. The above results were in accordance with the previous study [13], which indicated that LncRNA-XIST played an oncogenic role in NSCLC development and in line with the previous study [13].

Pyroptosis has recently been identified as a new pathway of cell death, which was closely related with cancer progression [22–24]. Notably, we found that knock-down of LncRNA-XIST increased pyroptosis associated biomarkers in A549 cells and induced pyroptotic cell death. Besides, our results showed that pyroptosis inhibitor NSA reversed the inhibiting effects of downregulated LncRNA-XIST on cell viability, which indicated that knock-down of LncRNA-XIST induced NSCLC cell death by triggering pyroptosis. In addition, ROS levels were increased by knocking down LncRNA-XIST in A549 cells. Similarly, ROS scavager NAC abrogated the inhibiting effects of downregulated LncRNA-XIST on cell viability, which suggested that knock-down of LncRNA-XIST also inhibited cell viability by inducing ROS generation. Previous studies have shown that pyroptosis could be induced by ROS generation [29, 31], and our results showed that NAC abrogated the effects of downregulated LncRNA-XIST on cell pyroptosis, which suggested that knock-down of LncRNA-XIST induced NSCLC cell pyroptosis by promoting ROS generation.

Oxidative stress could be modulated by multiple genes alteration [30]. By screening the differential expressions of anti-oxidant genes in NSCLC cells deficient or upregulation of LncRNA-XIST, SOD2 was filtered as the overlapping agent for investigation. Further results also validated that downregulated LnRNA-XIST promoted ROS production and cell pyroptosis by inhibiting SOD2 in NSCLC cells. The bioinformatics analysis showed that SOD2 co-expressed with other oxidative stress related genes, which were also related with LncRNA-XIST regulated oxidative stress in our experiments. However, the detailed mechanisms are still need to be elucidated. In addition, it has been reported that miRNAs were the bridge for the interactions between LncRNAs and its downstream targets [14, 15]. Based on that, we successfully proved that LncRNA-XSIT regulated SOD2 levels by targeting miR-335, which was in line with previous study [20]. Furthermore, our results showed that the effects of downregulated LncRNA-XIST on NSCLC cell proliferation, apoptosis and pyroptosis could be reversed by miR-335 inhibitor, which were all reversed by synergistically knocking down SOD2. The above results indicated that knock-down of LncRNA-XIST induced ROS generation, pyroptosis as well as apoptosis, and inhibited cell proliferation in NSCLC cells by regulating miR-335/SOD2 signal pathway.

Taken together, knock-down of LncRNA-XIST inhibited NSCLC cell proliferation and promoted cell apoptosis by triggering miR-335/SOD2/ROS signal pathway mediated pyroptotic cell death. Our study will provide potential therapeutic agents for NSCLC treatment in clinic.

## MATERIALS AND METHODS

### Human specimens

Human cancer specimens (N = 45) and paired adjacent normal tissues (N = 45) were collected from the patients diagnosed as NSCLC from The Third Affiliated Hospital of Harbin Medical University from 2013 to 2016. All the samples were collected and immediately refrigerated at −80 °C conditions for further experiments. The average age of NSCLC patients was 64 ± 8.83 years old, specifically, 20 of them below 65 years old and 25 of them beyond 65 years old. The gender ratio (male/female) of the participants was 35/10. All the patients has signed the inform consent. Besides, the involved clinical experiments were approved by the ethics committee of The Third Affiliated Hospital of Harbin Medical University. The detailed information of the patients in our study was summarized in Table 1.

**Table 1:** The clinical features of NSCLC patients involved in this research (N = 45).

**Parameters**	**Group**	**Total**
Age	≤65	20
	>65	25
Gender	Male	35
	Female	10
TNM Stage	I	8
	II	22
	III	7
	IV	8
Pathological Type	Squamous	14
	Adenocarcinoma	17
	Large Cell Lung Cancer	14
Smoking status	Non-smoker	5
	Somker	8
	Heavy smoker	7
Lymphatic Metastasis	No	19
	Yes	26

### Cell culture and vectors transfection

Human NSCLC cell lines (A549, H1299, SK-MES-1 and Calu-3) as well as human bronchial epithelial cell line (HBE) were purchased from American Type Culture Collection (ATCC, USA). All the cells were cultured in Roswell Park Memorial Institute 1640 medium (HyClone, Logan, UT) in the incubator with 37 °C and humidified 5% CO_2_ atmosphere. After the cell confluency reached about 60–80%, cells were prepared for further experiments. The sequence of LncRNA-XIST was amplified and cloned into pcDNA3.1 vector to construct LncRNA-XIST overexpression vectors pcDNA3.1-XIST (Sangon Biotech, Shanghai, China). The miR-335 mimic and inhibitor were designed and constructed by Sangon Biotech (Shanghai, China). The small interfering RNAs (siRNAs) specifically targeting LncRNA-XIST and SOD2 were purchased from Ribobio (Guangzhou, China). The above vectors were transfected into NSCLC cell lines (A549 and H1299) by Lipofectamine (Invitrogen, CA, USA) according to the manufacturer’s instructions.

### Bioinformatics analysis

The online StarBase software (http://starbase.sysu.edu.cn/index.php) was used to predict the binding regions of miR-335 and LncRNA-XIST. The online STRING software (https://string-db.org/) was employed to conduct the co-expression analysis among SOD2 and other anti-oxidant associated genes. Pan-cancer analysis (http://starbase.sysu.edu.cn/panCancer.php) was conducted to investigate the correlation of LncRNA-XIST with prognosis, miR-335 levels and SOD2 levels in lung adenocarcinoma patients.

### Real-Time qPCR

The total RNA of NSCLC tissues and cell lines were extracted by Trizol kit (Invitrogen, USA) according to the manufacturer’s instruction. The agarose gel electrophoresis (AGE) was performed to ensure no DNA contamination in the samples. After that, total RNA was reversely transcribed to cDNA by RT-PCR kit (Promega, USA). Real-Time qPCR kit (QIAGEN, Germany) was employed to quantify the mRNA levels of target genes. 

### Western Blot

The RIPA lysis buffer (Beyotime, China) was used extract total proteins from clinical tissues and cell lines. The proteins were separated by 10% sodium sulfate polyacrylamide gel electrophoresis (SDS-PAGE) and the targeted protein bands were transferred onto PVDF membrane (Sigma, USA). Subsequently, the PVDF membranes were probed with the primary antibodies including anti-β-actin (#ab8226, Abcam, USA), anti-SOD2 (#ab13534, Abcam, USA), anti-NLRP3 (#ab214185, Abcam, USA), anti-CyclinD1 (#ab16663, Abcam, USA), anti-Cyclin E2 (#ab32103, Abcam, USA), anti-CDK2 (#ab32147, Abcam, USA), anti-CDK4 (#ab108357, Abcam, USA), anti-CDK6 (#ab124821, Abcam, USA), anti-Caspase 3 (#ab13847, Abcam, USA), anti-Bax (#ab32503, Abcam, USA), anti-Bcl-2 (#ab185002, Abcam, USA), anti-Caspase 1 (#ab238979, Abcam, USA) and anti-IL-1β (#ab33774, Abcam, USA). After that, the membranes were incubated with anti-Rabbit IgG antibody at 4°C for 24h, and the enhanced chemiluminescent (ECL) system was employed to detect the protein bands. Finally, all the protein bands were quantified by Image J software. The primers sequences of the target genes were listed in Table 1.

**Table 2:** Primers sequences for Real-Time qPCR [13, 20].

**Gene**	**Primer sequences (strand)**
LncRNA-XIST	Forward: 5′-AATGACTGCCACTGCTGGG-3′
	Reverse: 5′-GTGTAGGTGGTTCCCCAAGG-3′
miR-335	Forward: 5′-TCAAGAGCAATAACGAAAAATGT-3′
	Reverse: 5′-GCTGTCAACGATACGCTACGT-3′
SOD1	Forward: 5′-AAGGCCGTGTGCGTGCTGAA-3′
	Reverse: 5′-GGCCCACCGTGTTTTCTGGA-3′
SOD2	Forward: 5′-CCCAGATAGCTCTTCAGCCTGCACT-3′
	Reverse: 5′-TAAGCGTGCTCCCACACATCAATCC-3′
SOD3	Forward: 5′-TCGTCCTCTTCCGGCAGCTT-3′
	Reverse: 5′-GCTTCTTGCGCTCTGAGTGCT-3′
GPX1	Forward: 5′-GCGGGGCAGGTACTACTTA-3′
	Reverse: 5′-CTCTTCGTTCTTGGCGTTCT-3′
GPX2	Forward: 5′-ATTTGGACATCAGGAGAACTGT-3′
	Reverse: 5′-CTTCAGGTAGGCGAAGACA-3′
GPX3	Forward: 5′-GCCGGGGACAAGAGAAGT-3′
	Reverse: 5′-GAGGACGTATTTGCCAGCAT-3′
GPX7	Forward: 5′-AACTGGTGTCGCTGGAAAG-3′
	Reverse: 5′-AAACTGGTTGCAGGGGAAG-3′
FOXO3	Forward: 5′-GTGCGGTGCGTGCCCTACTT-3′
	Reverse: 5′-GCTCTTCCAGTGCCTTCGT-3′
Nrf2	Forward: 5′-GACCTAAAGCACAGCCAACACAT-3′
	Reverse: 5′-CTCAATCGGCTTGAATGTTTGTC-3′
U6	Forward: 5′-CTCGCTTCGGCAGCACA-3′
	Reverse: 5′-AACGCTTCACGAATTTGCGT-3′

### Enzyme-linked immunosorbent assay (ELISA)

NSCLC cell lines were cultured under the standard conditions and the supernatants were collected by centrifugation at 1500 rpm for 10 min. The expression levels of IL-18 in the supernatants were detected by the ELISA kit (#abz15539, Abcam, USA) following the protocol. Briefly, 50 μL of the above samples were added to each well of the 96-well plates, and 50 μL antibody cocktail was further added to the wells and incubated at room temperature for 1h. After that, TMB substrate (100μL) was added to each well for 10 min and 100μL stop solution was used to stop the reactions. Finally, a Sector 6000 plate reader was used to quantify IL-18 levels and read optical density (OD) values at 450nm.

### Colony formation assay

The NSCLC cells were diluted to the concentration of 1000 cells / 200μL and seeded into the 6-well plates at the density of 1000 cells/well. The plates were then placed at the incubator at 37 °C with humidified 5% CO_2_ atmosphere for 14 days and stained with 0.1% crystal violet (Beyotime, Shanghai) for 1h to visualized the colonies according to the manufacturer’s instruction. After that, the plates were photographed and the colony numbers were counted by a gel documentation system (BioRad, USA).

### MDA assay

Malondialdehyde (MDA) has been proved to be the degraded product of polyunsaturated lipids, which could be used to measure the level of lipid peroxidation and oxidative stress. MDA could react with thiobarbituric acid as a thiobarbituric acid reactive substances (TBARS) to form a 1:2 MDA-TBA adduct, hence measuring the contents of TBARS reflected MDA levels. The MDA assay kit (Beyotime Biotechnology, Shanghai, China) was used to evaluate TBARS according to the manufacturer’s instruction.

### L-012 dye staining

LncRNA-XIST was knocked-down in A549 cells and overexpressed in H1299 cells respectively, L-012 dye was then employed to detect extracellular NADPH oxidase-derived superoxide to reflect the extent of oxidative stress according to the manufacturer’s instruction. Briefly, A549 and H1299 cells were cultured under the standard conditions and diluted to the concentration of 4–6 × 10^4^ cells/well into 96-well plates (Thermo, USA) in phenol free DMEM medium. After that, 500 μM of L-012 was added and incubated with the cells for 10 min, the luminescence was detected by a Gemini EM microplate reader (Molecular Devices, USA) at excitation wavelength of 488 nm and emission wavelength of 525 nm respectively.

### Dihydroethidium (DHE) staining

DHE staining was next used to detect intracellular ROS levels in A549, H1299 and HBE cells. In brief, the NSCLC cells were incubated with 10 μM of DHE for 30 min at 37°C without light exposure. After that, cells were then washed with phosphate buffer saline (PBS) for 3 times. DM500 fluorescence microscope purchased from Olympus Coporation (Japan) was employed to capture 10 random fields from each sample at the magnification of 400×. After that, the positive areas were analyzed and the fluorescence intensity was quantified by using Image J analysis software.

### Dual-luciferase reporter gene system

The fragments of LncRNA-XIST were amplified by PCR and cloned into a pmirGLO Expression Vector (Sangon Biotech, China) to generate XIST-wild-type reporter vector (XIST-wt). Similarly, the corresponding mutant LncRNA-XIST fragments were also inserted into the vectors to form XIST-mut. The above vectors, miR-335 mimics and NC were transfected into 293T cells by Lipofectamine (Invitrogen, CA, USA). Finally, the Dual-Luciferase Reporter Assay System (TransGene, China) was employed to detect the luciferase activity.

### Flow cytometry (FCM)

The FITC-Annexin V apoptosis Detection Kit (YEASEN corporation, China) was used to detect cell apoptosis ratio according to the protocol. In brief, the NSCLC cells were double stained with propidium iodide (PI) and Annexin V for 30 mins in darkness. Cell apoptosis ratio was then detected by using a FACSCalibur Flow Cytometer (Becton Dickinson, USA).

### Statistical analysis

All the data were collected and represented as Mean ± SD (Standard deviation). The statistical significance was analysed by SPSS 18.0 software (IBM Corporation, Armonk, NY). Statistical differences between two groups were determined using Student’s t-test and among multiple groups were analysed by using analysis of variance (ANOVA). The Pearson analysis was conducted to analyse the co-expression of multiple genes in NSCLC tissues collected from clinic. A two-tailed *P* < 0.05 was considered statistical significance. All the images were drawn by GraphPad Prism software (Version 7.0, La Jolla, CA, USA).

## Supplementary Material

Supplementary Figure 1
