# Supplementary material for: Downregulation of LncRNA-XIST inhibited development of non-small cell lung cancer by activating miR-335/SOD2/ROS signal pathway mediated pyroptotic cell death
Source: Aging (Albany NY). 2019 Sep 25;11(18):7830–46. doi: 10.18632/aging.102291 (PMC6781979; doi:10.18632/aging.102291)
Supplement: Supplementary Figure 1 [file aging-11-102291-s001.pdf]

SUPPLEMENTARY FIGURE

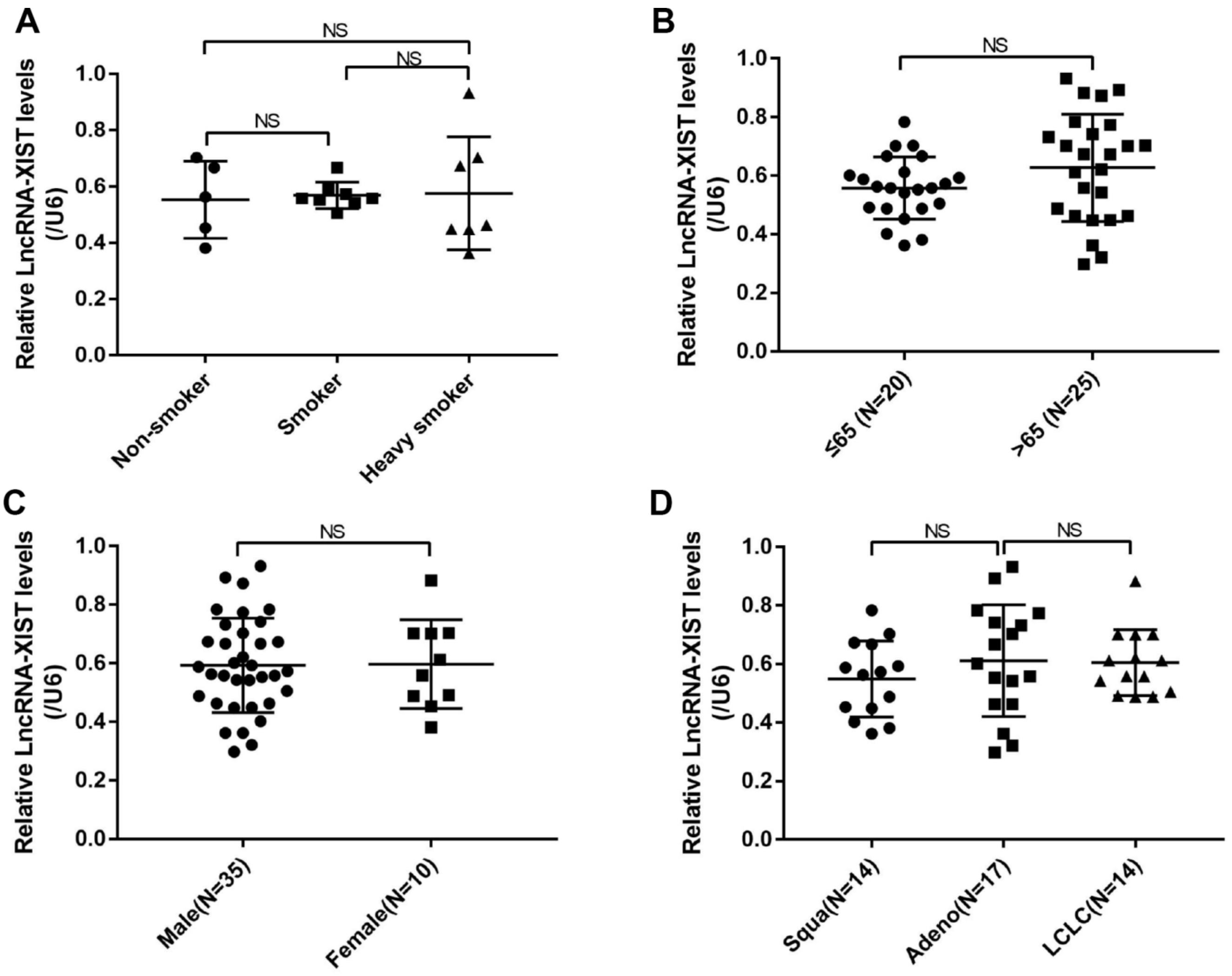

Supplementary Figure 1. The expression levels of LncRNA-XIST in patients with different (**A**) smoking status, (**B**) age, (**C**) gender and (**D**) pathological type. ("NS" represented no statistical significance, "*" represented $p < 0.05$, "**" represented $p < 0.01$).
